# Catalytic Dehydration of Fructose to 5-Hydroxymethylfurfural in Aqueous Medium over Nb_2_O_5_-Based Catalysts

**DOI:** 10.3390/nano11071821

**Published:** 2021-07-13

**Authors:** Elisa I. García-López, Francesca Rita Pomilla, Bartolomeo Megna, Maria Luisa Testa, Leonarda Francesca Liotta, Giuseppe Marcì

**Affiliations:** 1 Department of Biological, Chemical and Pharmaceutical Sciences and Technologies (STEBICEF), University of Palermo, Viale delle Scienze, 90128 Palermo, Italy; elisaisabel.garcialopez@unipa.it; 2 “Schiavello-Grillone” Photocatalysis Group, Department of Engineering, University of Palermo, Viale delle Scienze, 90128 Palermo, Italy; francesca.pomilla@unimib.it (F.R.P.); Bartolomeo.megna@unipa.it (B.M.); 3 Institute of Nanostructured Materials (ISMN)-CNR, Via Ugo La Malfa, 153, 90146 Palermo, Italy; marialuisa.testa@cnr.it (M.L.T.); leonardafrancesca.liotta@cnr.it (L.F.L.)

**Keywords:** biomass valorization, 5-HMF, heterogeneous acid catalysis, niobium oxide, titanium oxide, green chemistry

## Abstract

The catalytic dehydration of fructose to 5-hydroxymethylfurfural (HMF) in water was performed in the presence of pristine Nb_2_O_5_ and composites containing Nb and Ti, Ce or Zr oxides. In all experiments, fructose was converted to HMF using water as the solvent. The catalysts were characterized by powder X-ray diffraction, scanning electron microscopy, N_2_ physical adsorption, infrared and Raman spectroscopy and temperature-programmed desorption of NH_3_. Experimental parameters such as fructose initial concentration, volume of the reacting suspension, operation temperature, reaction time and amount of catalyst were tuned in order to optimize the catalytic reaction process. The highest selectivity to HMF was ca. 80% in the presence of 0.5 g·L^−1^ of bare Nb_2_O_5_, Nb_2_O_5_-TiO_2_ or Nb_2_O_5_-CeO_2_ with a maximum fructose conversion of ca. 70%. However, the best compromise between high conversion and high selectivity was reached by using 1 g·L^−1^ of pristine Nb_2_O_5_. Indeed, the best result was obtained in the presence of Nb_2_O_5_, with a fructose conversion of 76% and a selectivity to HMF of 75%, corresponding to the highest HMF yield (57%). This result was obtained at a temperature of 165° in an autoclave after three hours of reaction by using 6 mL of 1 M fructose suspension with a catalyst amount equal to 1 g·L^−1^.

## 1. Introduction

**Scheme 1:**
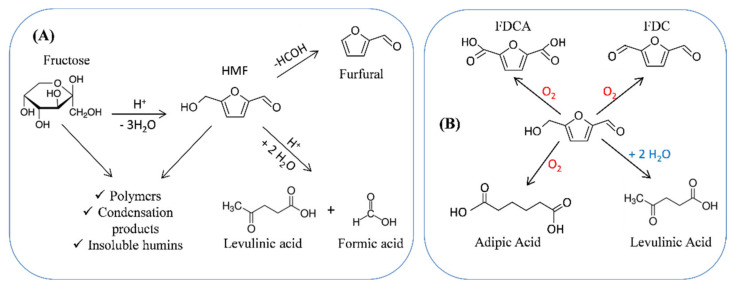
(**A**) Typical reaction scheme for HMF formation from fructose dehydration in acidic catalytic conditions; (**B**) HMF as platform molecule to obtain some added-value compounds in aerobic condition.

Biomass is a green renewable resource easily available and considered a precious local good. In this century, the conversion of biomasses into valuable fuels or chemicals has been largely investigated [1]. Hexoses, such as glucose, fructose or xylose, are the most abundant monosaccharides present in biomass and their acid-catalyzed dehydration can give rise to a variety of platform chemicals. For instance, the acid-catalyzed dehydration of fructose gives rise to 5-hydroxymethylfurfural (HMF), which is converted to equimolar amounts of levulinic acid (LA) and formic acid, as shown in Scheme 1A. HMF production requires the removal of three water molecules of the fructose molecule, although, in the first steps, less dehydrated intermediate species could suffer intermolecular condensation, producing polymers and insoluble humins as side products (Scheme 1A). Moreover, 5-Hydroxymethylfurfural (HMF) can be converted to various chemical products and liquid fuels, potentially useful to obtain fine chemicals, pharmaceuticals, furanose-based polymers and bio-polymeric plastic materials [2,3]. 

The partial oxidation of HMF, as shown in Scheme 1B, yields 2,5-furandicarboxyaldehyde (FDC) [4], a high-added-value aldehyde, or 2,5-furandicarboxylic acid (FDCA), which represents an alternative to terephthalic acid in PET (PolyEthylene Terephthalate) manufacturing and could give rise also to adipic acid, extensively used in nylon preparation [5]. The hydration of HMF can yield levulinic acid, an important intermediate for polymer production [6,7].

Due to the great potential of HMF as a versatile platform molecule, its production has been approached by different methodologies [8], trying to minimize side reactions and/or HMF further transformation. Although many endeavors have been devoted to HMF production from glucose or fructose in water, organic solvents or ionic liquids, the intrinsic reaction efficiency is still low [9,10,11,12,13,14]. A brown coloration of the reaction mixtures is generally observed, indicating the formation of polymeric bio-products, i.e., humins [15]. The use of dimethylsolfoxide (DMSO), an aprotic polar solvent, gives rise to high yields of HMF; however, the high affinity of HMF for DMSO requires large amounts of extraction solvent [8,16]. In polar protic solvents, such as methanol or ethanol, the decomposition rate of HMF to levulinic acid and formic acid was considerably lower than in water, as well as the amount of humins produced. Protic solvents, particularly sec-butanol, were found to be a better option than aprotic ones to yield HMF from fructose [17].

The undesirable formation of dark-colored side products, tarry carbonaceous solids called humins, is a drawback in the dehydration and hydrolysis of acid-catalyzed glucose or fructose, especially when compared to enzymatic processes. These oligomeric materials possess a complex structure and composition containing furan rings and carbonyl groups conjugated with carbon–carbon double bonds [18,19]. At the moment, the most likely applications for these humins are either as a fuel or to form by catalytic pyrolysis liquid products (humin oil) enriched in phenolics and aromatics [20].

Kinetic studies on the decomposition of fructose in aqueous homogeneous solutions in the presence of H_2_SO_4_ (5 mM to 1 M concentrations) demonstrated that, at low temperatures, the formation of humins decreased, thus increasing the yield of HMF [21,22].

Despite the advantage of using water as an environmentally benign solvent, the chemistry in aqueous systems suffers from relatively low HMF yields because of subsequent reactions to levulinic acid and humins, as evidenced in Scheme 1. However, the tuning of the experimental conditions could reduce the formation of undesirable products. The presence of mineral acids such as HCl, H_2_SO_4_, HNO_3_ or H_3_PO_4_ [23,24] is typically associated with high toxicity, corrosion, difficulty of separation and recovery of the catalysts along with its poisoning. Heterogeneous acidic catalysts overcome most of the disadvantages of mineral acids, offering easy separation from reaction products, the possibility of recycling the catalyst and a higher selectivity than that obtained in the presence of homogeneous acidic substances [23]. Few solid acids can be considered acceptable as catalysts for fructose dehydration in aqueous media to obtain HMF, in terms of activity, stability and insolubility at high temperature and pressure. The literature reports heterogeneous acidic catalysts for this reaction, such as resins [12], ZrO_2_ [25,26,27] and TiO_2_ [27], aluminum–molybdenum mixed oxides [28], WO_3_ [26] or Nb_2_O_5_-based composites [8,28,29,30,31,32,33,34], zeolites [35], sulfonic acid-functionalized mesoporous materials [36,37] supported catalyst Nafion-resin-functionalized mesocellular silica foam [38] or mesoporous acidic solids [39].

Transition metal oxides such as Nb_2_O_5_, ZrO_2_, WO_3_ or TiO_2_ typically possess Lewis and Brønsted acid sites that allow a heterogeneous pathway reaction for a wide set of reactions such as fructose dehydration to obtain HMF.

Among the acidic transition metal oxides, Nb_2_O_5_-based materials have given promising results as catalysts for fructose conversion to HMF. Nb_2_O_5_ is one of the most used acidic catalysts and it has been largely studied for dehydration [40,41] and also in catalytic reactions requiring acidic solids, such as glycerol acetylation [42]. In fact, for its acidic character, the hydrated niobium pentoxide (Nb_2_O_5_·nH_2_O) is also known as niobic acid.

Nb_2_O_5_, both bare and in the presence of Ce, Ti and Zr, has been used for catalytic fructose dehydration. Stosic et al. suggested that the fructose conversion and selectivity to HMF were closely related to the amount of strong acid sites present on the surface of the catalyst [31,32]. Such materials were active in the dehydration of fructose in water to levulinic acid and HMF. For experiments lasting 10 h, the conversions were in the range 70–90% and the selectivity values to HMF from 25 to 35%.

Yang et al. used Nb_2_O_5_·nH_2_O for the same reaction in a water/2-butanol biphasic system at 160 °C for 50 min and in the presence of 2 g·L^−1^ of catalyst, obtaining an HMF yield of 89% [28]. Niobic acid and niobium phosphate at temperatures from 90 to 110 °C and pressures from 2 to 6 bar in aqueous medium and a continuous reactor were employed to perform the fructose dehydration in HMF, with a conversion equal to ca. 45% [33]. The complete fructose conversion with a yield of 86% in HMF was reached in the presence of a home-prepared Nb_2_O_5_ using DMSO as the solvent at 400 °C [34]. Dehydration of fructose with a conversion of 94% and yield to HMF equal to 85% was obtained at 180 °C after 3 h of reaction in hot compressed water in the presence of Nb_2_O_5_ [43]. The literature often reports that the use of bare Nb_2_O_5_ is associated with various drawbacks, such as low catalytic activity, poor reusability and extreme experimental conditions, which limit its application. The strategy of preparing mixed metal oxide catalysts seemed to give promising results, enhancing the acidic strength, increasing the specific surface area and strengthening the stability of these materials in comparison with pristine Nb_2_O_5_, for various organic reactions [21,31,32,39]; however, acidic mixed metal oxides based on Nb_2_O_5_ have been seldom used in the dehydration of hexoses to obtain HMF.

In this research, pristine and composites based on Nb_2_O_5_, containing Ce, Zr or Ti oxides, were prepared. These materials were used as catalysts in fructose dehydration in aqueous medium to obtain HMF. The catalytic behavior was investigated by modifying some experimental parameters in the reacting system, such as: (i) initial fructose concentration, (ii) reaction temperature, (iii) reaction time and (iv) amount of catalyst. Consequently, the aim of this research was to explore the possibility to increase the catalytic performance of Nb_2_O_5_ by preparing and testing composite materials based on Nb_2_O_5_ and CeO_2_ or ZrO_2_ or TiO_2_ and, moreover, to tune all the operative parameters in order to optimize the conversion of fructose and its selectivity towards HMF formation.

## 2. Materials and Methods

### 2.1. Preparation of the Pure and Mixed Metal Oxide

A mesoporous niobium oxide was prepared by adding, under vigorous stirring, 4.4 mmol of NbCl_5_ to a solution containing 1 g of triblock copolymer surfactant F127 in 25 mL of ethanol. The same procedure was followed to prepare the Nb oxide-based composites in the presence of other metals. For the preparation of these solids, 4.4 mmol of NbCl_5_ salt was added to the same amount of F127 in ethanol solution, which contained also dissolved metal salts (ZrCl_4_, CeCl_3_ or TiCl_4_). In any case, the niobium/metal molar ratio was equal to 0.5. Water (6 mL) was successively added to the solution containing the metal chloride and the obtained white suspension was maintained under stirring for 18 h at 40 °C. The resulting solid was hence dried overnight at 60 °C and then transferred into a ceramic crucible. The powder was calcined in static air atmosphere at 500 °C for 3 h, using a heating ramp of 0.1 °C·min^−1^. The resulting solids were mechanically ground in a mortar and labeled: Nb_2_O_5_, Nb_2_O_5_-ZrO_2_, Nb_2_O_5_-CeO_2_ and Nb_2_O_5_-TiO_2_. Furthermore, a pure TiO_2_ powder was also prepared by using TiCl_4_ as precursor, following the same procedure but in the absence of NbCl_5_.

### 2.2. Characterization of the Catalysts

The crystalline structure of the calcined samples was determined by powder X-ray diffraction patterns (XRD), performed on a Bruker D5000 diffractometer equipped with a Kristalloflex 760 X-ray generator (Bruker AXS GmbH, Karlsruhe, Germany) and with a curved graphite monochromator using Cu Kα radiation (40 kV/30 mA). The data were recorded in a 2θ range of 20°-80° with a step size of 0.05° and time per step of 20 s. The crystalline phases of samples were analyzed according to ICSD (Inorganic Crystal Structure Database) files. The mean crystallite size was calculated by the Debye–Scherrer equation: D = 0.9λ/Bcosθ, where D represents the average crystalline size, 0.9 is the Scherrer parameter, λ is the wavelength of the X-ray radiation (0.15406 nm), B denotes the full width at half maximum of the peak (FWHM), and θ is the angular position of the peak.

Bulk and surface characterizations were carried out in order to investigate the physical–chemical properties of the powders. Scanning electron microscopy (SEM) was performed using an FEI Quanta 200 ESEM microscope (FEI Company, Hillsboro, Oregon) operating at 20 kV on specimens upon which a thin layer of gold had been evaporated. An electron microprobe used in energy dispersive mode (EDAX) was employed to obtain information on the actual metals’ ratio present in the samples. The specific surface areas (SSA), pore volume and pore size of the materials were measured by N_2_ adsorption–desorption isotherms using a Micromeritics ASAP2020 system (Micromeritics Instrument Corp., Norcross, GA, USA). Before analysis, the samples were degassed in vacuum at 250 °C for 2 h; then, the measurement was performed at liquid nitrogen temperature (−196 °C). The Brunauer–Emmett–Teller (BET) method was used to calculate the SSA. The Barrett–Joyner–Halenda (BJH) method was applied to the desorption branch to estimate the pore volume and pore size distribution.

Infrared spectra of the used samples in KBr Sigma Aldrich (Darmstadt, Germania) pellets were obtained with an FTIR–8400 Shimadzu (Shimadzu Europa GmbH, Albert-Hahn-Str. 6-10, Duisburg, Germany) spectrophotometer and recorded with 4 cm^−1^ resolution and 256 scans. Raman spectra were recorded on powdered samples packed into sample cups. Spectra were registered by using a Renishaw (Wotton-under-Edge, Glouchestershire, UK) in-via Raman spectrometer equipped with an integrated microscope and with a charged-coupled device (CCD) camera. A He/Ne laser operating at 632.8 nm was used as the exciting source Wotton-under-Edge, Glouchestershire, UK). The power of the laser used was 15% of the maximum value, which was around 300 mW.

The acidity of the calcined materials was determined by temperature-programmed desorption of ammonia (NH_3_-TPD) experiments performed on a Micromeritics Autochem 2910 apparatus equipped with an ultraviolet gas analyzer (ABB, Limas 11 (ABB S.p.A. Sesto San Giovanni, Milano, Italy). The sample amount of 0.3 g was pre-treated in He flow at 100 °C for 30 min. Then, after cooling to room temperature, ammonia adsorption was performed by flowing a stream of 5% NH_3_/He (30 mL·min^−1^) for 1 h. In order to remove all the physically adsorbed ammonia, the sample was purged by flowing He (100 mL·min^−1^) at 100 °C for 1 h. Then, after cooling to room temperature, ammonia desorption started by flowing He (30 mL·min^−1^) and heating up to 600 °C (rate of 10 °C·min^−1^), holding time at 600 °C for 30 min. The ammonia concentration profiles, ppm of NH_3_ desorbed/g_cat_ under He flow (30 mL·min^−1^), were plotted versus time and temperature by using data registered with the ultraviolet gas analyzer ABB, Limas 11 (ABB S.p.A. Sesto San Giovanni, Milano, Italy). The total amount of desorbed NH_3_ [mmol NH_3_∙g_cat_^−1^] was then calculated by the integration of the ammonia concentration profiles versus time.

### 2.3. Catalytic Experiments

The fructose dehydration reactions were carried out in a 50 mL Teflon covered backer placed in a stainless-steel autoclave. The reactor contained a fructose aqueous solution in a concentration range from 0.1 to 1 M. Preliminary fructose dehydration tests were carried out in homogeneous regime at natural pH (5.5) and in the presence of H_2_SO_4_ at pH equal to 2.7. The amount of catalyst was, where not stated otherwise, 2 g·L^−1^. The volume of the suspension was 6 mL, where not stated otherwise. The reaction temperature’s influence was investigated from 100 to 165 °C. In order to heat the reacting suspension at the desired temperature, the autoclave was immersed in a heated oil bath. The effects of reaction time and amount of catalyst were also explored from 1 to 5 h and 0.5 to 2 g·L^−1^, respectively.

The compounds in the reaction mixture were identified and quantified by analysis of the solution through injection on Thermo Scientific Dionex Ultimate 3000 HPLC (Thermo Fisher Scientific, Waltham, MA, USA) equipped with a diode array and refractive index detectors. The column was a REZEK ROA Organic acid H+ Phenomenex and the eluent an aqueous 2.5 mM H_2_SO_4_ solution with a flow rate equal 0.6 mL min^−1^. Retention times and UV spectra of the compounds were compared with those of standards purchased from Sigma-Aldrich with a purity of >99%. The catalytic performance in terms of percentage of conversion of fructose (X), selectivity towards HMF formation (S) and HMF yield (Y) was defined as follows:

X = ([fructose]_i_ − [fructose]_f_)/[fructose]_i_·100 (1)

S = [HMF]/([fructose]_i_ − [fructose]_f_)·100 (2)

Y = [HMF]/[fructose]_i_·100 (3)

where [fructose]_i_ and [fructose]_f_ are the initial and the final molar concentration of fructose, respectively, and [HMF] is the HMF molar concentration at the end of the experiment.

## 3. Results and Discussion

### 3.1. Bulk and Textural Metal Oxide Characterization

**Figure 1:**
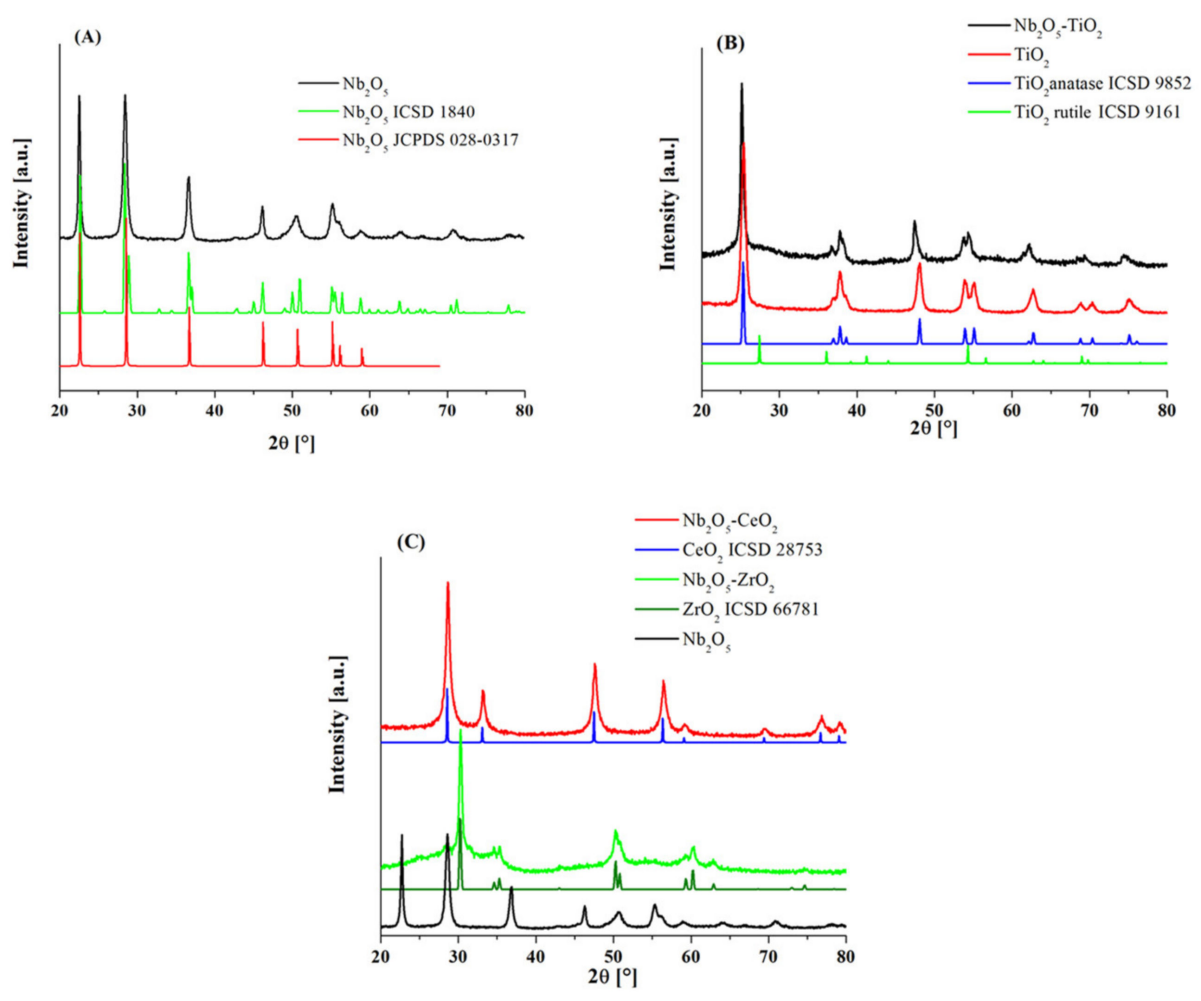
XRD patterns of (**A**) bare Nb_2_O_5_ and ICSD/JCPDS reference files; (**B**) Nb_2_O_5_-TiO_2_, TiO_2_ and anatase and rutile ICSD reference files; (**C**) Nb_2_O_5_-CeO_2_, Nb_2_O_5_-ZrO_2_ and the corresponding ICSD reference files.

In order to investigate the crystalline structure of the prepared materials, XRD patterns were registered. Niobium oxide, Nb_2_O_5_, is reported to exist in different polymorphic forms, and the phase transformations of niobium oxide strongly depend on the calcination treatment [44]. The bare Nb_2_O_5_ powder shows defined diffraction peaks that can be attributed to an orthorhombic structure (ICSD reference n. 1840, space group Pbam); see Figure 1A. The same structure was detected for Nb_2_O_5_ nanorods prepared by the hydrothermal method [45].

Other articles reported for Nb_2_O_5_ calcined at T< 500 °C a pseudohexagonal structure called the TT-Nb_2_O_5_ phase (ICDD no. 00-028-0317, lattice constants: c = 3.94 Å and a = 3.64 Å (space group: P6/mmm); then, after increasing the temperature up to 600 °C, the structure became orthorhombic [46]. On the other hand, the orthorhombic (T-Nb_2_O_5_) and pseudohexagonal (TT-Nb_2_O_5_) phases are difficult to distinguish in terms of structure. The T phase is thought to be simply a more ordered structure of the TT phase [47]. By comparing our experimental pattern of Nb_2_O_5_ with two reference files (ICDD n. 00-028-0317 and ICSD n.1840), a better correspondence with the orthorhombic structure was found after considering the diffraction peaks at 2θ angles in the 40–60° range (see Figure S1A in the supporting information). According to the primary reference [48], signals related to the Nb_2_O_5_ phase (ICDD no. 00-028-0317) are reported up to 2θ of 70°.

The X-ray diffractograms of the pristine TiO_2_ and Nb_2_O_5_-TiO_2_ sample are displayed in Figure 1B along with the ICSD references for anatase (n. 9852, tetragonal, space group I 41/amdS) and rutile (n. 9161, tetragonal, space group P42/mnm). Peaks of the anatase phase were identified in the diffractograms of bare TiO_2_ and Nb_2_O_5_-TiO_2_, although in the latter sample, the titania features appear poorly defined suggesting a disordered structure. The mean crystallite size was estimated from Scherrer’s equation applied to the most intense peak (the 101) and the crystallite sizes for the anatase phase in the Nb_2_O_5_-TiO_2_ (in the range 17.0 nm) were larger than those for the bare TiO_2_ (11.6 nm).

No signals ascribable to the rutile phase or peaks of Nb-containing oxides were detected in the above-mentioned patterns. According to the literature [49], Ti(IV) in the anatase structure is six-coordinated and has an ionic radius equal to 0.605 Å, while Nb (V), also six-coordinated, has an ionic radius of 0.64 Å; therefore, the doping of the anatase by the Nb(V) cations likely occurs and these phenomena can justify the absence of Nb_2_O_5_ crystalline features [50]. Comparable results have been reported for Nb-doped TiO_2_, with up to 38 at% of Nb, showing the existence of only pure anatase in the XRD pattern for samples calcined up to 550 °C [51].

The enlargement of the XRD pattern, for 2θ between 20° and 40° (see Figure S1B in the supporting information), clearly evidences a shift to lower angles of the (101) from 25.4° in the pure TiO_2_ to 25.15° in the Nb_2_O_5_-TiO_2_ sample, in accordance with Nb insertion into the lattice of the anatase structure. A similar shift was observed for the 004 planes of the Nb-doped titania along with a visible perturbation of the peak symmetry. In Figure 1C, the XRD patterns of Nb_2_O_5_-CeO_2_ and Nb_2_O_5_-ZrO_2_ are plotted and compared with CeO_2_ (ICSD n. 28753, cubic, space group F m −3 m) and ZrO_2_ (ICSD n. 66781, tetragonal, space group P 42/n m c Z) references. The diffractogram of bare Nb_2_O_5_ is also reported for comparison. The XRD diffractogram of the Nb_2_O_5_-CeO_2_ sample shows only the diffraction peaks typical of the fluorite structure of CeO_2_, with no evidence of other phases. Analogous results were obtained also by Stosic [31,32] and references therein, where it is reported that the CeO_2_ affects the crystallization of niobia. A detailed analysis of the XRD pattern, in the 2θ range 20–40° (see Figure S1C in supporting info), reveals a shift of the (111) peak of the CeO_2_ fluorite structure from 28.55° to slightly higher angles, 28.69°, which is indicative of an insertion of Nb(V), having an ionic radius 0.74 Å smaller than 0.97 Å for eight-coordinated Ce(IV) [49], into the ceria lattice. A similar shift was detected for the (200) peak.

As far as the Nb_2_O_5_-ZrO_2_ sample is concerned, as reported in Figure 1C, the diffraction peaks are compatible with the tetragonal ZrO_2_ crystalline structure and no other features attributable to Nb_2_O_5_ phases are visible. Assuming that Nb(V) is eight-coordinated with an ionic radius of 0.74 Å and, since Zr(IV) is six-coordinated in the tetragonal system with an ionic radius equal to 0.72 Å, both values are very similar, so we can assume that if Nb enters the ZrO_2_ lattice, no modification occurs. In all of the Nb_2_O_5_-based mixed oxides, the presence of amorphous niobium oxide, not detectable by XRD, cannot be excluded.

**Figure 2:**
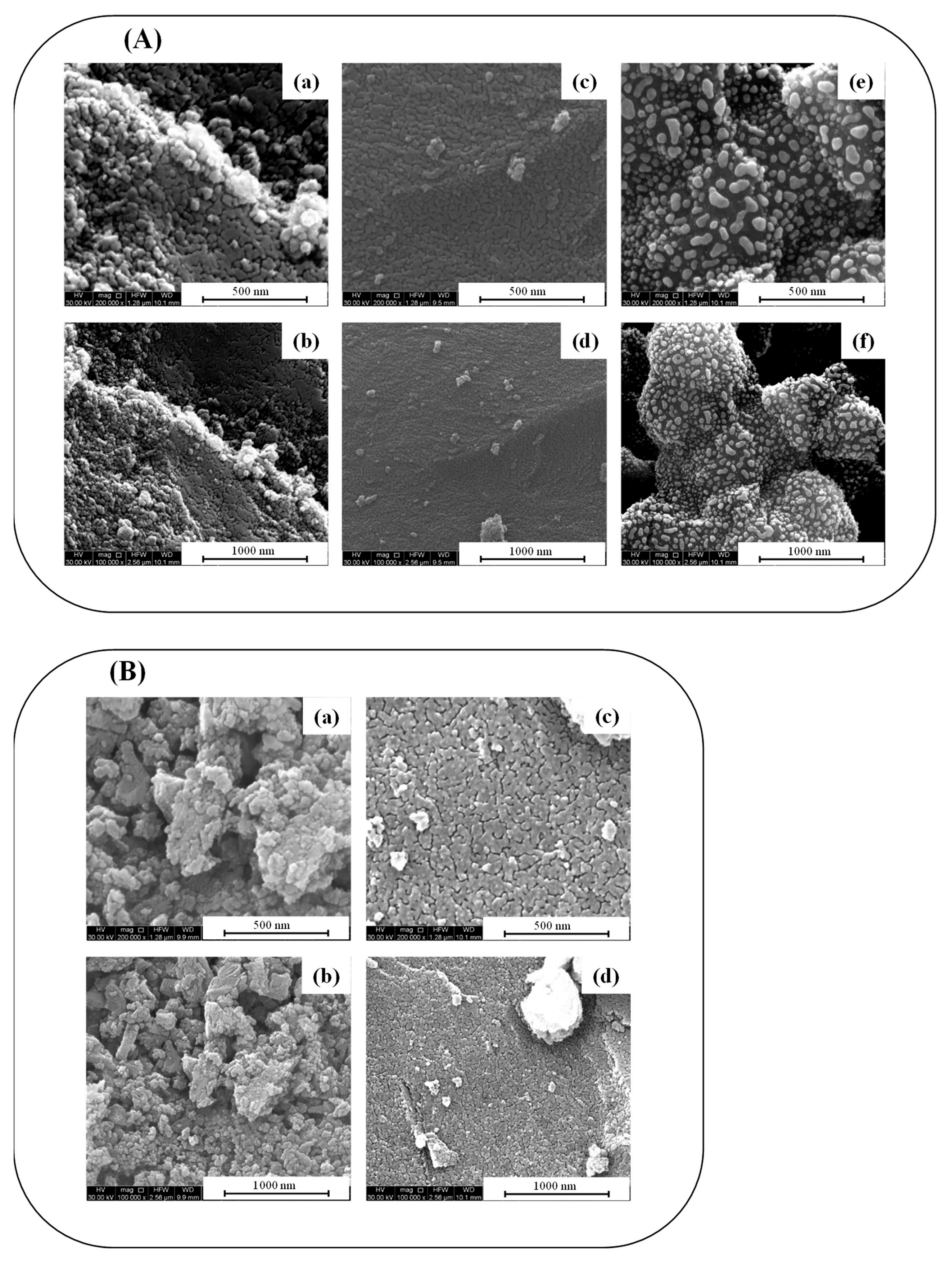
SEM micrographs of each prepared powder at two different magnifications, 10^5^ and 2·10^5^: (**A**) bare Nb_2_O_5_ (**a**,**b**), Nb_2_O_5_/TiO_2_ (**c**,**d**) and bare TiO_2_ (**e**,**f**) and (**B**) Nb_2_O_5_/CeO_2_ (**a**,**b**) and Nb_2_O_5_/ZrO_2_ (**c**,**d**).

The SEM micrographs shown in Figure 2A,B are useful to study the morphology of the investigated samples. Figure 2A reports the micrographs of bare Nb_2_O_5_ and TiO_2_ and the micrographs of the Nb_2_O_5_/TiO_2_ composite material at two different magnifications. The morphology of the Nb_2_O_5_ and Nb_2_O_5_/TiO_2_ samples is similar; indeed, the particles of these materials are formed by the agglomeration of grains interconnected to each other. The size of these grains is larger in the case of bare Nb_2_O_5_ (ca. 50 nm) with respect to that of the Nb_2_O_5_/TiO_2_ sample (ca. 20 nm). It is interesting to note that the sizes of the voids between the various grains are very similar to those of the pores measured through the adsorption of N_2_, and this will be discussed later. They are around 10 nm in the case of Nb_2_O_5_ and around 5 nm for Nb_2_O_5_/TiO_2_. On the other hand, the morphology of bare TiO_2_ is completely different, indicating that the presence of amorphous Nb_2_O_5_, albeit as a minority component in the Nb_2_O_5_/TiO_2_, is able to define the morphology of the composite. This fact was evident also for the other two composites, whose micrographs are reported in Figure 2B. Indeed, the morphology of Nb_2_O_5_/CeO_2_ and Nb_2_O_5_/ZrO_2_ is similar to that of bare Nb_2_O_5_, as is the size of the grains constituting the particles, which are only slightly larger (ca. 60 nm). Moreover, in these cases, the sizes of the voids between the various grains are very similar to those of the pores measured through the adsorption of N_2_ (ca. 10 nm for Nb_2_O_5_/CeO_2_ and ca. 5 nm for Nb_2_O_5_/ZrO_2_). Moreover, the Nb_2_O_5_/CeO_2_ presents also some macropores. As far as the atomic ratios of niobium/(Ti, Ce or Zr) are concerned, Table 1 reports both nominal values and those measured by EDX analysis. The obtained figures indicate that only in the case of Nb_2_O_5_/TiO_2_ did the EDX results confirm the nominal value of 0.5. For the other composites, we observed a higher content of niobium in comparison with the nominal one; this fact can be explained by assuming a partial superficial segregation of Nb_2_O_5_, as can be inferred from XRD characterization, not revealing any crystalline feature of niobium oxide.

**Figure 3:**
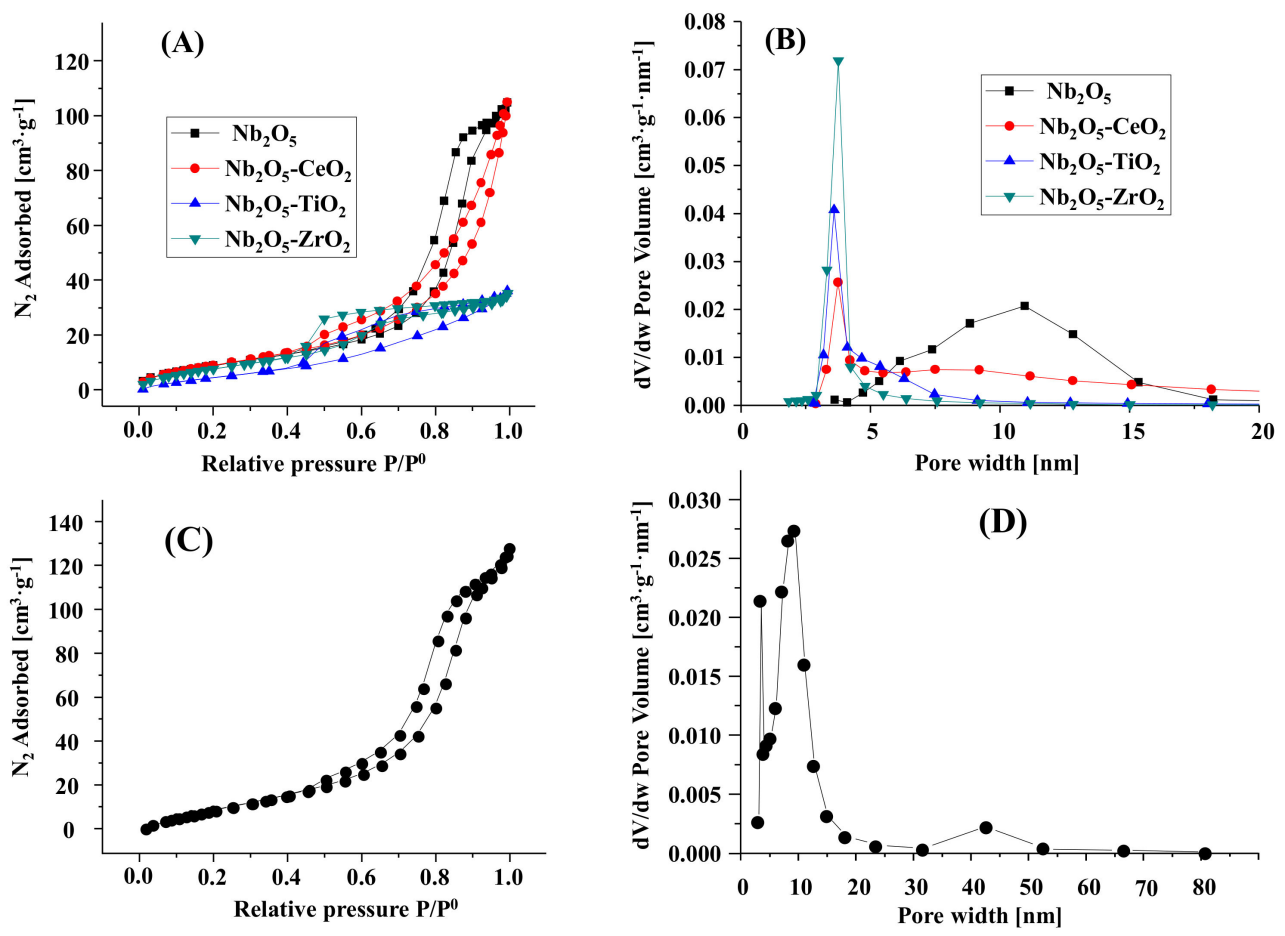
(**A**) N_2_ adsorption–desorption isotherms and (**B**) pore size distribution as dV/dw vs. pore width calculated by BJH method on the desorption branch for Nb_2_O_5_ and Nb_2_O_5_-based mixed oxides as well as (**C**,**D**) for the bare TiO_2_.

The N_2_ adsorption–desorption isotherms and the corresponding pore size distributions of the investigated Nb_2_O_5_-based catalysts as well as for the bare TiO_2_ are shown in Figure 3. Values of the specific surface area (SSA) and porosity are listed in Table 1. The isotherms are classical type IV, as defined by IUPAC, with hysteresis typical of mesoporous materials. The SSA value of bare Nb_2_O_5_ is equal to 54 m^2^·g^−1^; comparable values were registered for the Nb_2_O_5_-CeO_2_ and slightly lower for Nb_2_O_5_-ZrO_2_. A drop in surface area was observed for the sample Nb_2_O_5_-TiO_2_, with a value of only 38 m^2^·g^−1^, and the decrease appears even more pronounced in comparison with the SSA of pure TiO_2_, which is as high as 73 m^2^·g^−1^. Therefore, it seems that the presence of Nb_2_O_5_ (around 45 wt%) strongly affects, as already discussed in the SEM section, the morphological properties of the TiO_2_. Pore volume and pore size distribution values change as a function of the material composition as it results from the hysteresis shape and pore size distribution. The pure Nb_2_O_5_ was characterized by a broad pore size distribution centered at around 10 nm, with a cumulative pore volume equal to 0.17 cm^3^·g^−1^. Very narrow and centered at around 5 nm is the pore distribution for Nb_2_O_5_-ZrO_2_ and Nb_2_O_5_-TiO_2_; concurrently, a significant decrease in the pore volume occurred in comparison with bare Nb_2_O_5_. The Nb_2_O_5_-CeO_2_ was characterized by a narrow peak at around 3 nm along with a pronounced and broad shoulder centered at ~ 8–10 nm, with a mean pore size distribution equal to 10 nm and cumulative pore volume of 0.17 cm^3^·g^−1^. As far as pure TiO_2_ is concerned, in line with the high SSA value (73 m^2^·g^−1^), a relatively high pore volume was measured (0.21 cm^3^·g^−1^) and the corresponding mean pore size was centered at 9 nm. A direct comparison with Nb_2_O_5_-based compounds is difficult, due to the different nature of the materials; however, it is confirmed that the presence of Nb oxide strongly influences the morphological properties of the Nb_2_O_5_-TiO_2_ system.

**Figure 4:**
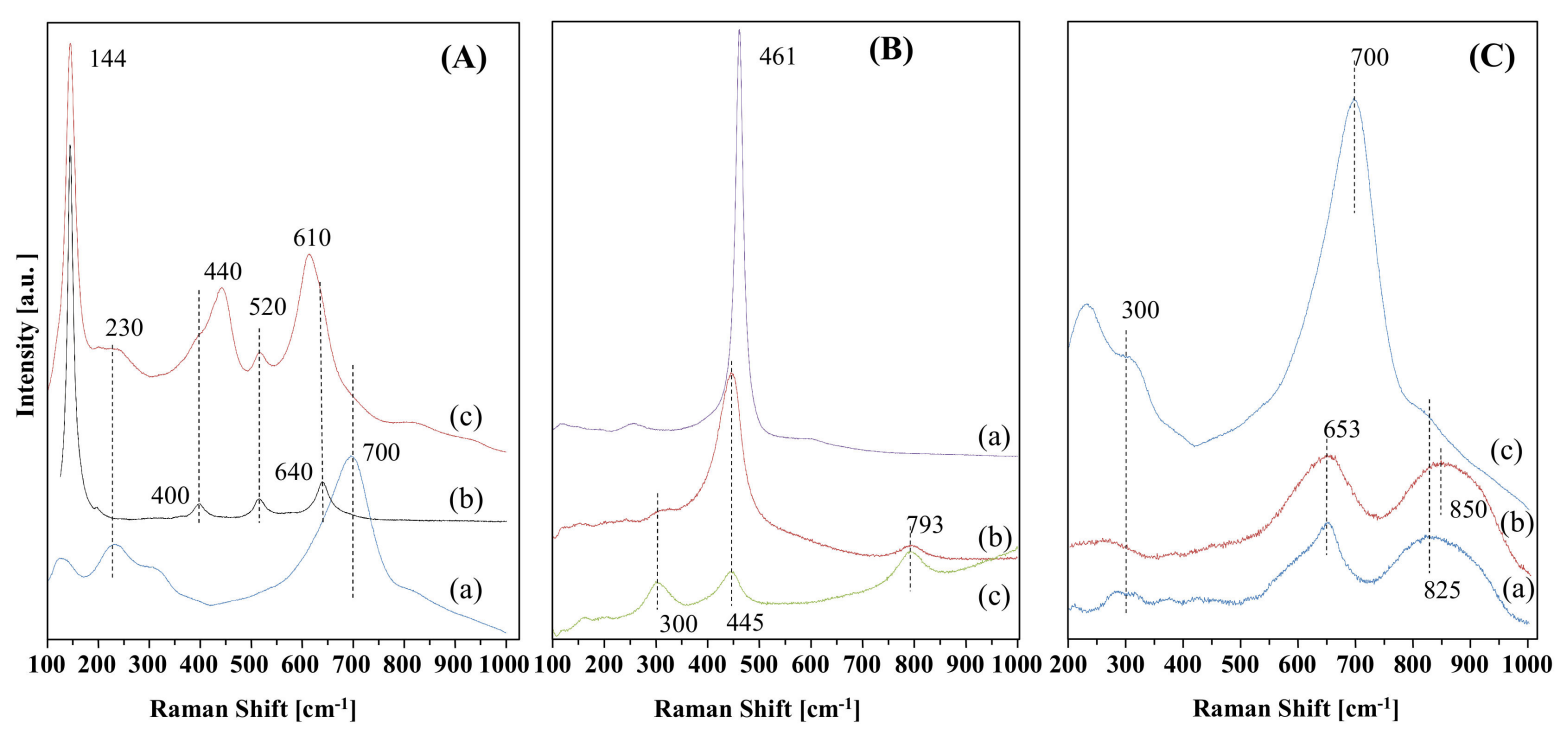
Raman spectra of the samples: (**A**) (a) pristine Nb_2_O_5_, (b) bare TiO_2_ and Nb_2_O_5_-TiO_2_ binary material; (**B**) three different measures on the same sample Nb_2_O_5_-CeO_2_ binary material and (**C**) (a) and (b) two different measures on the same sample Nb_2_O_5_-ZrO_2_ binary material and (c) pristine Nb_2_O_5_.

Raman spectroscopy was used to further investigate the structural features of the prepared samples as Raman spectroscopy is a very sensitive technique to determine the phase composition in transition metal oxides. Each measurement was performed at different positions on the surface of the powders in order to verify the homogeneity of the material. The spectra obtained for all of the samples are reported in Figure 4. Both bare Nb_2_O_5_ and TiO_2_ along with the composite Nb_2_O_5_-TiO_2_ are reported in Figure 4A. The Raman spectrum of the pristine Nb_2_O_5_ shows a broad band centered at 700 cm^−1^, typical for all niobium oxides, and assigned to the vibrations of the Nb–O–Nb bridges of slightly distorted octahedral NbO_6_ connected by sharing corners and associated with Brønsted acid centers [31,44]. Jehng and Wachs attributed the band to an orthorhombic Nb_2_O_5_ phase [44]. This result is in agreement with the XRD investigation. The niobium oxide structures are composed of NbO_6_ octahedra; however, when increasing the amorphous degree of the structure, slightly distorted NbO_6_, NbO_7_ and NbO_8_ polyhedra are also present [52] and this could be the reason for the broadening of the band centered at 700 cm^−1^. A weak and broad signal around 900 cm^−1^ assigned to the symmetric stretching mode of Nb=O surface sites has been also reported for niobia. These species, present in small concentrations, could be due to the Raman experiment itself because of the heating of the sample by the laser [44] and could explain the widening of the spectrum and the lack of a good baseline. A group of weak and broad bands in the low-wavenumber region (200–300 cm^−1^) was assigned to the bending modes of the Nb–O–Nb linkages [44]. The pristine TiO_2_ spectrum reports peaks located at 144, 400, 520 and 640 cm^−1^, which are characteristic of the anatase polymorph [53], in agreement with the XRD results. The vibrational properties of the Nb_2_O_5_-TiO_2_ sample are different from that of the bare TiO_2_, even if the transitions assigned to anatase are present in the spectrum. It is worth remembering that Ti and Nb ions are similar in size, and Nb(V), occupying preferentially the octahedral sites available on the anatase, can give rise to important deformations of the structure responsible for the shifting of the Raman bands of the Nb_2_O_5_-TiO_2_ with respect to the bare TiO_2_. As has been previously stated, the radius of Nb(V) (0.640 Å) is slightly larger than that of Ti(IV) (0.605 Å); therefore, a solid solution can be formed, confirming the XRD findings (see Figure S1B). The Nb(V) cation, inducing stress in the TiO_2_ lattice, may hinder the growth of TiO_2_ crystallite. In the Nb_2_O_5_-TiO_2_ material, the formation of substitutional defects could also induce oxygen vacancies in comparison to the pristine TiO_2_. A further feature in the Raman spectrum of Nb_2_O_5_-TiO_2_ is a broad peak located at around 610 cm^−1^. This band has been attributed to Nb–O–Nb vibrations in amorphous Nb_2_O_5_. The broadness of this transition in amorphous Nb_2_O_5_ is ascribed to the distribution of NbO_6_ and NbO_7_, and NbO_8_ polyhedra [54]. Interestingly, the Nb_2_O_5_-TiO_2_ Raman spectrum lacks the characteristic band active modes at 700 cm^−1^ corresponding to Nb_2_O_5_, so it can be concluded that, if this oxide was present, it would be amorphous. The Raman data reported so far are in good agreement with the XRD characterization.

As mentioned above, the micro-Raman analysis allowed us to study the heterogeneity of the samples because it was possible to record spectra on different positions of the sample by focusing the microscope on different areas. Indeed, the recorded Raman spectra of the Nb_2_O_5_-CeO_2_ sample showed three different Raman patterns, reported in Figure 4B, evidencing the heterogeneity of its structure. The spectrum reported in Figure 4B (a) can be ascribed to a CeO_2_ phase exhibiting a single peak at 461 cm^−1^. The literature reports that the F2g mode due to the symmetrical stretching of Ce-O vibrational unit in eightfold coordination in the cubic fluorite structure of pristine CeO_2_ occurs at 463 cm^−1^ [55]. The shift observed by us with respect to the reported value could be attributed to a lattice distortion because of the incorporation of a smaller cation, such as Nb(V), in the CeO_2_ fluorite structure, in agreement with XRD data showing a shift of the (111) and (200) peaks of the CeO_2_ in the Nb_2_O_5_-CeO_2_ sample to slightly higher angles with respect to the ICSD reference (see Figure S1C). The other two spectra recorded for the Nb_2_O_5_-CeO_2_ sample are similar to each other, showing a transition at 445 cm^−1^ attributed to Ce-O symmetrical stretching in a distorted lattice.

Two different Raman patterns of the Nb_2_O_5_-ZrO_2_ sample are reported in Figure 4C, along with that of the bare Nb_2_O_5_. According to the XRD results, a distorted tetragonal ZrO_2_-like could be present so various Raman-active modes would be expected: 148, 164, 266, 322, 339, 467, 609 and 642 cm^−1^ [56]. Both Nb and Zr are effectively present; consequently, we cannot exclude the formation of a solid solution. In previous reports, the Raman spectra of zirconia doped with niobium samples obtained after calcination at 500 °C did not show any Raman-active mode, despite the presence of crystalline phase(s) detected by XRD [57]. Indeed, according to Li et al., when the ZrO_2_ phase is present in the bulk of particles, as revealed by XRD, it could be surrounded by a layer of amorphous material, which would render the oxide undetectable by Raman in the wavelength used in this study [58]. Regarding the band observed for Nb_2_O_5_-ZrO_2_ at 653 cm^−1^ in Figure 4C, it could be attributed to Nb–O–Nb vibrations in amorphous Nb_2_O_5_ [54], analogously to that which appears in Figure 4A for the Nb_2_O_5_-TiO_2_ sample.

**Figure 5:**
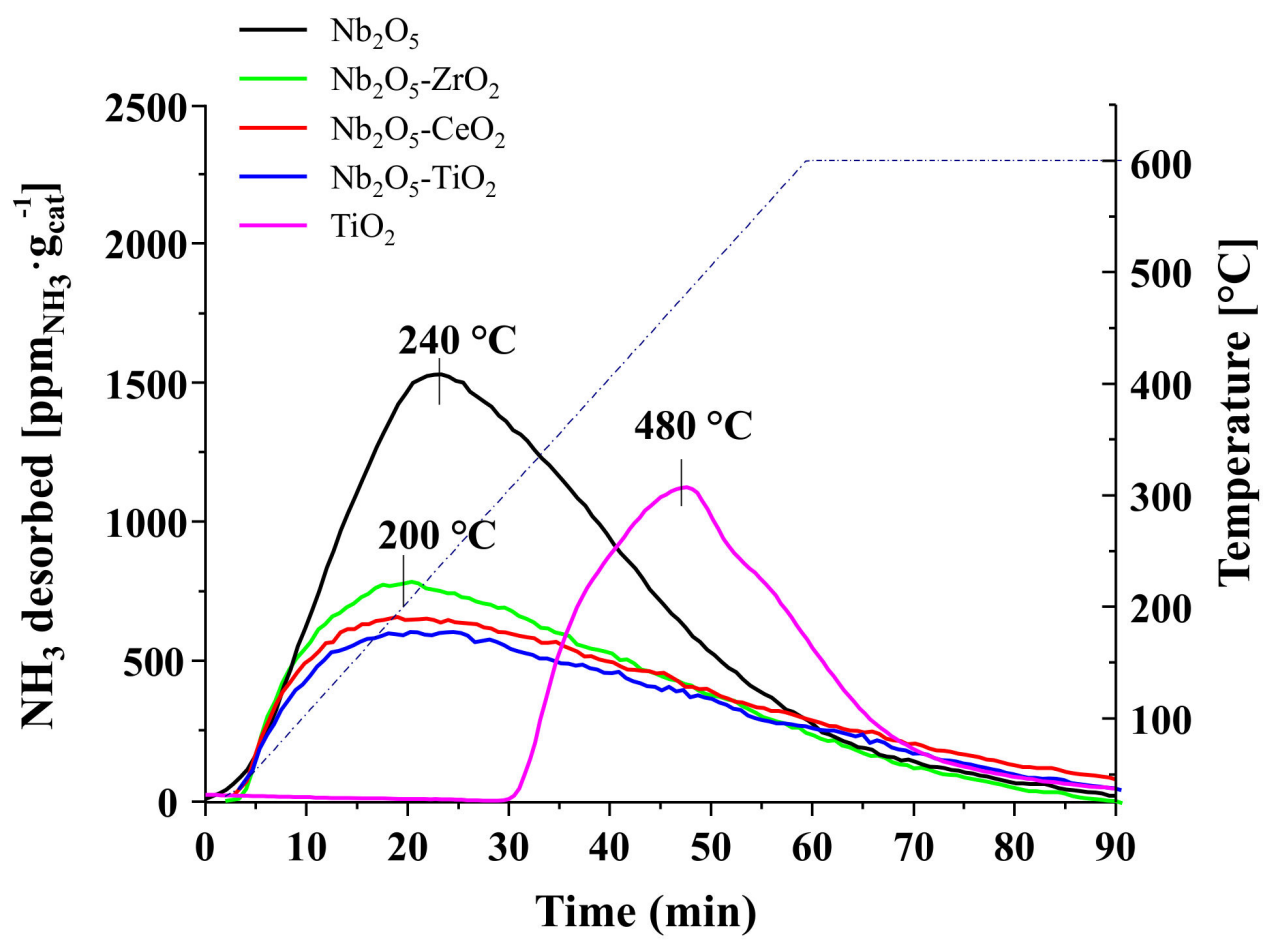
NH_3_-TPD curves of the samples registered versus time by heating from room temperature up to 600 °C (rate of 10 °C·min^−1^), holding time at 600 °C for 30 min.

The acidic properties of bare Nb_2_O_5_ and Nb_2_O_5_ mixed oxide samples were evaluated by NH_3_-TPD experiments carried out from room temperature up to 600 °C. The strength of the catalytic acid surface sites can be classified by the desorption temperature of NH_3_ upon heating the sample under an inert atmosphere. According to the literature on Nb_2_O_5_ catalysts, the acidic strength distribution can be classified into the following regions: 80–150 °C physisorbed NH_3_; the desorption of NH_3_ in the range of 150–250 °C describes weak acid sites, 250–350 °C medium acid sites and above 350 °C corresponds to strong acid sites [59,60]. Figure 5 displays the ppm of NH_3_ desorbed per gram of catalyst as a function of the time and temperature of the experiment. In Table 2, the acidity values derived by TPD are listed in terms of the total amount of desorbed NH_3_ [mmol NH_3_∙g_cat_^−1^].

For all of the Nb_2_O_5_-based catalysts, NH_3_ desorption started to occur from a temperature below 150 °C, suggesting the presence of some very weak adsorbed NH_3_, or some residual physisorbed species. Desorption of ammonia above 150 °C indicated the presence of weak, medium and strong acid sites. On the contrary, in the case of bare TiO_2_, only the presence of medium and strong acid sites was observed. Bare Nb_2_O_5_ was characterized by an intense broad peak, whose area corresponds to 0.076 mmol NH_3_∙g_cat_^−1^. Such a peak centered at 240 °C was attributed to weak and medium acid sites. Pure TiO_2_ showed an ammonia desorption peak equal to 0.055 mmol NH_3_∙g_cat_^−1^ with a maximum at 480 °C, which is the typical temperature of strong acidic sites. The desorption profiles of the Nb_2_O_5_-ZrO_2_, CeO_2_ and TiO_2_ mixed oxides show similar features, all being characterized by a desorption peak centered at a slightly lower temperature (~200 °C) than the bare Nb_2_O_5_ and corresponding to a lower amount of acid sites (0.038–0.043 mmol NH_3_∙g_cat_^−1^).

All peaks related to both pristine Nb_2_O_5_ and the mixed oxides are asymmetric, with a tailed shape up to 450–500 °C, suggesting that some strong acid sites also contribute to the overall acidity. The lower acidity of niobia mixed oxides is consistent with the lower weight content of Nb_2_O_5_ per g of catalyst. Moreover, the presence of medium and of some strong acidic sites, desorbing in the range of temperature typical of the bare Nb_2_O_5_, confirms the segregation of amorphous niobium oxide on the surfaces of the mixed catalysts, but can be also ascribed to the formation of new Lewis acid sites arising from the formation of lattice defects induced by the insertion of Nb (V) into the TiO_2_, CeO_2_ and ZrO_2_ lattice. On the other hand, the lower acidity of Nb_2_O_5_-TiO_2_ with respect to the pristine oxides further confirms that the structure of Nb_2_O_5_ is not retained in the mixed oxide, according to XRD and Raman characterization.

### 3.2. Catalytic Fructose Dehydration Reactivity

Preliminary tests were carried out in a homogenous regime both at natural (5.5) and acidic pH (2.7) by adding H_2_SO_4_ to the fructose solution (1 M) heated for 3 h at 165 °C. The conversion was 100% in the presence of H_2_SO_4_, whereas at natural pH, it was 65%. The selectivity to HMF in H_2_SO_4_ was slightly lower (35%) than that obtained at the natural pH (43%). Experiments carried out at the natural pH by decreasing the initial fructose concentration, 0.3 and 0.1 M, gave rise to lower conversions of 31% and 23%, respectively, whereas the selectivity to HMF increased to 79% and 84% for 0.3 and 0.1 M initial fructose concentrations, respectively. The lower selectivity to HMF at the higher fructose concentration can be explained by considering the greater intermediate agglomeration/condensation side reactions to form insoluble polymers and humins (see Scheme 1). Indeed, the higher the initial fructose concentration, more turbid and dark brown becomes the suspension color after the run, due to the polymeric species formed.

The presence of Nb_2_O_5_ heterogeneous catalyst enhanced the conversion of fructose in comparison to the experiments carried out in the homogeneous regime at natural pH. The conversion of fructose in the presence of Nb_2_O_5_ is nearly equal to that attained in the homogeneous regime in the presence of H_2_SO_4_ at pH 2.7, although the selectivity to HMF increased from 35 to 43%. Further experiments carried out in the presence of Nb_2_O_5_ by decreasing the reacting temperature from 165 to 140 and 100 °C conclude that the decrease in reacting temperature gave rise also to a decrease in both conversion and selectivity (see Table 3). The yield of the reaction, reported in Table 3, is indicative of the presence of side or further products, different from HMF, as shown in Scheme 1.

**Figure 6:**
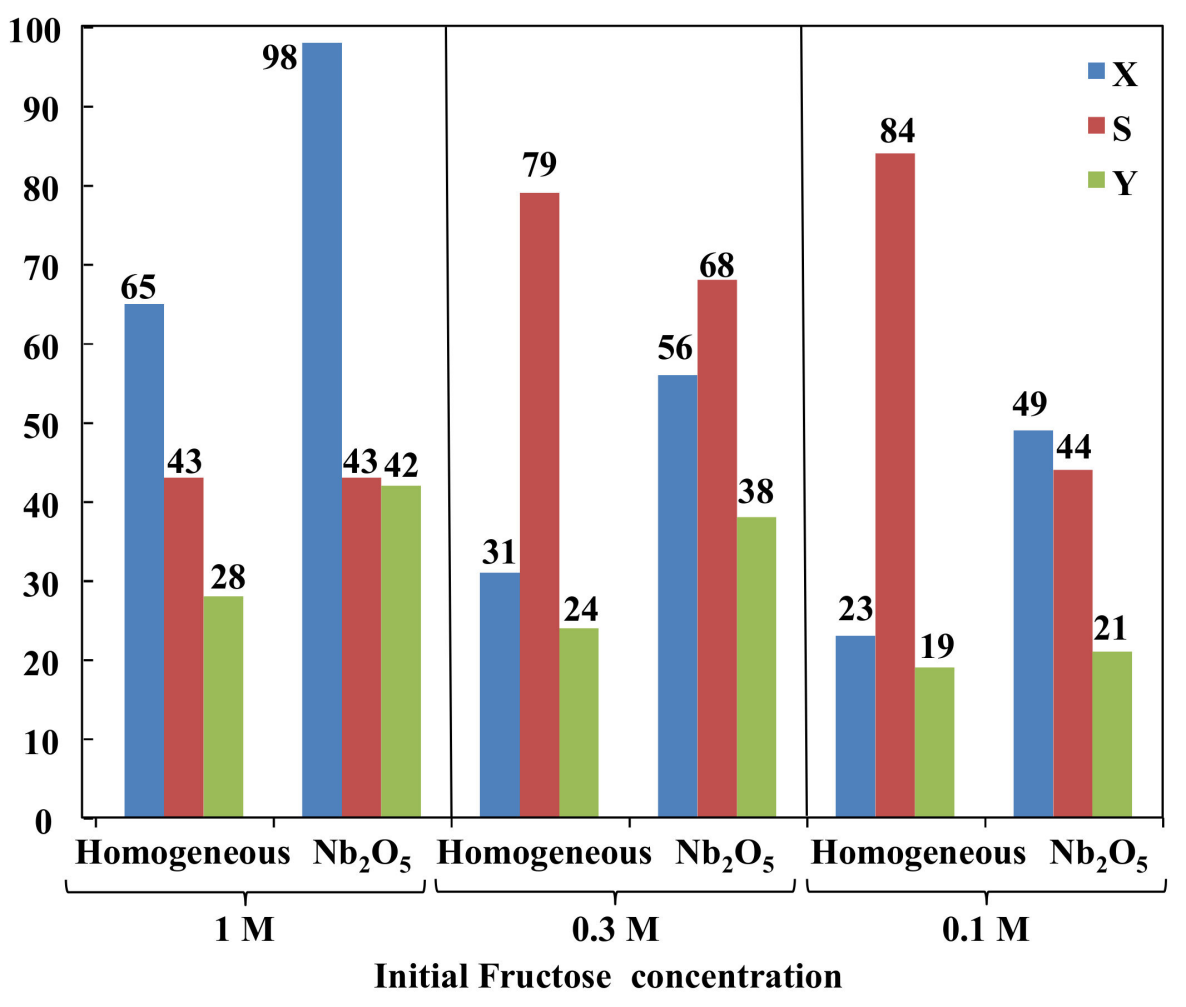
Fructose conversion (**X**), selectivity to HMF (**S**) and yield (**Y**) in different reactive conditions, i.e., in homogeneous regime at natural pH as well as in the presence of Nb_2_O_5_ by using different initial concentration of fructose. Experiments were carried out at 165 °C for 3 h by using 6 mL of fructose suspension. In the heterogeneous catalytic experiments, the Nb_2_O_5_ amount was 2 g·L^−1^.

The catalytic fructose conversion in the presence of Nb_2_O_5_ was studied also by modifying the initial concentration of the monosaccharide, as reported in Figure 6. A perusal of Figure 6 indicates that both in homogeneous and in the presence of Nb_2_O_5_, the conversion of fructose increases when increasing the initial sugar concentration. On the contrary, the selectivity to HMF generally increased by decreasing the initial fructose concentration.

The presence of Nb_2_O_5_, despite significantly increasing the fructose conversion, did not change the selectivity to HMF with respect to the homogeneous run when the initial fructose concentration was 1 M but reduced it for the tests carried out by using the other two concentrations. Moreover, and surprisingly, by reducing the initial fructose concentration from 0.3 to 0.1 M, its dehydration selectivity to HMF in the presence of Nb_2_O_5_ decreased from 68 to 44%. This insight shows that in order to maximize the fructose conversion or the selectivity to HMF, the initial fructose concentration solution should be 1 or 0.3 M. It is worth noting that the HMF yield was nearly the same for the experiments carried out at 1 M or 0.3 M fructose initial concentration (ca. 40%), whereas by working with a 0.1 M concentration, the yield almost halved to 21%.

**Figure 7:**
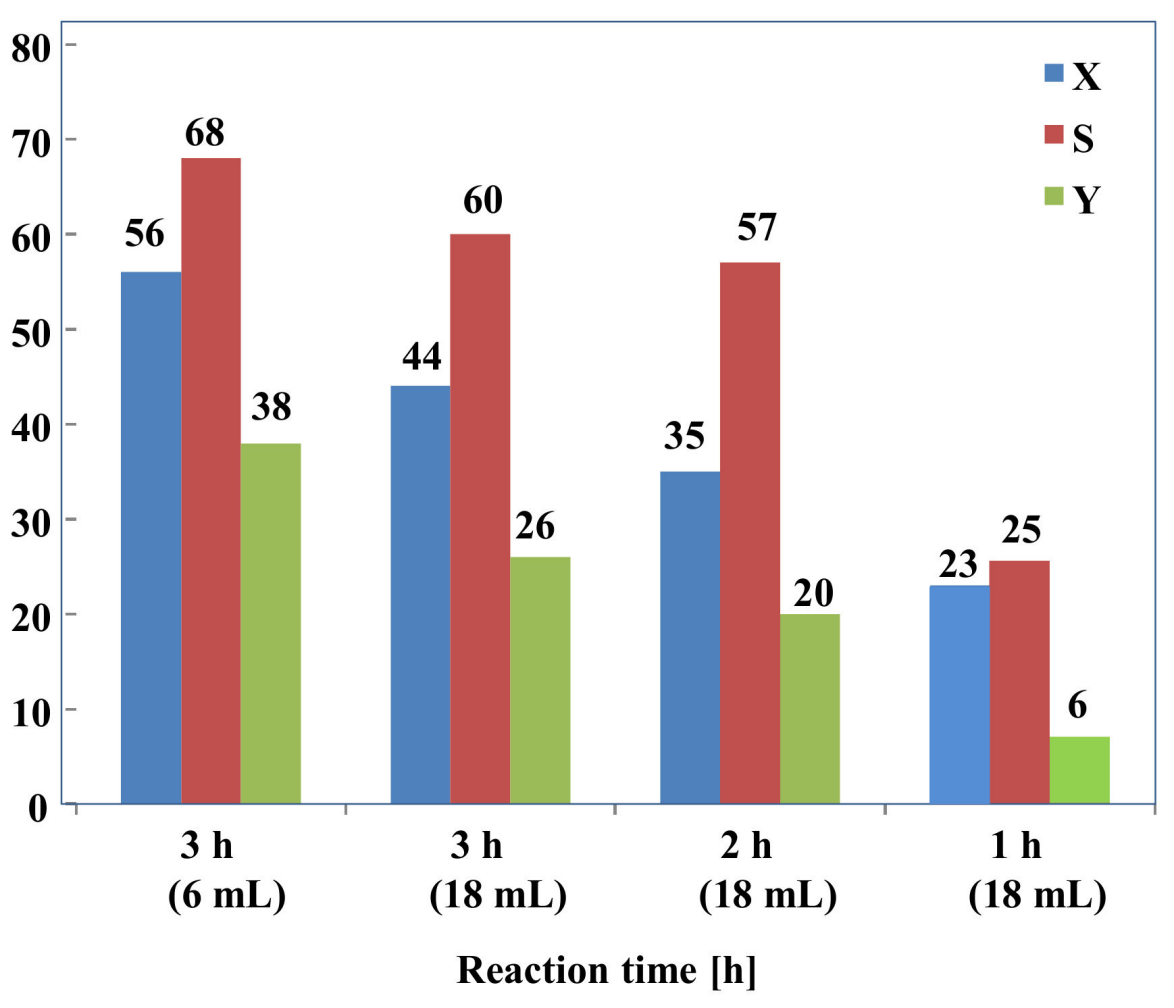
Fructose conversion (**X**), selectivity to HMF (**S**) and yield (**Y**) obtained in the catalytic experiments carried out at 165 °C by using an initial fructose concentration of 0.3 M and in the presence of Nb_2_O_5_ (2 g·L^−1^).

The amount of reacting suspension was studied also as a parameter to optimize the operative conditions. Some experiments were carried out enhancing the total volume from 6 mL (used in the runs reported above) to 18 mL. The initial concentration of fructose was 0.3 M in the presence of 2 g·L^−1^ of Nb_2_O_5_. The obtained results, for experiments lasting from 1 to 3 h, are reported in Figure 7.

**Figure 8:**
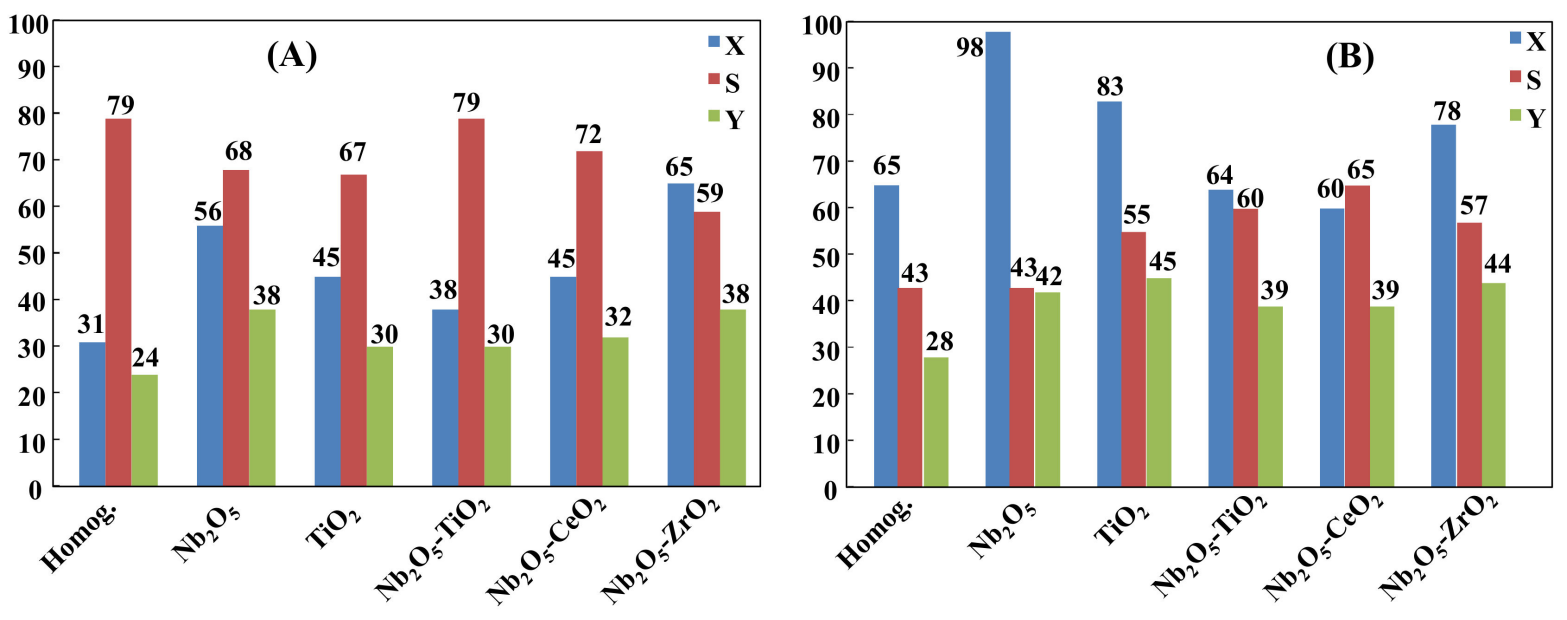
Fructose conversion (**X**), selectivity to HMF (**S**) and yield (**Y**) obtained in the experiments carried out with an initial fructose concentration of 0.3 M (**A**) and of 1.0 M (**B**) at 165 °C for 3 h in the presence of 6 mL of the reaction mixture containing an amount of solid, in the heterogeneous catalytic experiments, of 2 g·L^−1^.

The performance of the system after 3 h of reaction was lower by using 18 mL of suspension instead of 6 mL. This fact can be explained by considering the different time needed to reach the final temperature inside the reaction suspension. Indeed, in any case, the autoclave was immersed in an oil bath maintained at the desired temperature and the reaction time was measured starting from this point. Consequently, since it took a longer time to reach the final temperature of 165 °C, the suspension of 18 mL remained at this temperature for a shorter time with respect to the suspension of 6 mL. This fact can also explain why, by reducing the reaction time to 2 h or even to 1 h, the conversion and selectivity are even lower (see Figure 8).

From the above-reported screening, the best operative conditions for the current catalytic set-up resulted to be a temperature of 165 °C, a volume of the reaction mixture of 6 mL and a catalytic reaction lasting 3 h for an initial fructose concentration from 0.3 M to 1 M. By using these operational parameters, also the other heterogeneous catalysts prepared were tested and their activity compared for the fructose dehydration reaction. Two sets of experiments were carried out with an initial concentration of fructose of 0.3 or 1 M. The results obtained are reported in Figure 8.

For an initial monosaccharide concentration of 0.3 M (Figure 8A), the presence of the heterogeneous catalysts was found to be beneficial in terms of conversion, and, although the selectivity generally slightly decreased, a higher yield with respect to the homogeneous reaction was observed. The results obtained in the presence of pristine TiO_2_ were slightly lower than those obtained with Nb_2_O_5_. By comparing the results obtained in the presence of Nb_2_O_5_-TiO_2_ and Nb_2_O_5_-CeO_2_ with those obtained by using the bare metal oxides, the use of the former resulted in a slightly higher selectivity to HMF but with a lower yield, at least with respect to that shown in the presence of bare Nb_2_O_5_. The Nb_2_O_5_-ZrO_2_ catalyst gave rise to the higher conversion of fructose with a lower selectivity and nearly the same yield in HMF than that obtained with the bare Nb_2_O_5_.

The increasing trend of fructose conversion is not in agreement with the higher acidity measured by TPD of NH_3_. Indeed, the most acidic material was the bare Nb_2_O_5_ but the most active catalyst in terms of conversion was Nb_2_O_5_-ZrO_2_, although its acidity was lower than that of Nb_2_O_5_ and similar to that of the other catalysts. These insights indicate that the acidity is not the only parameter influencing the conversion of fructose and the selectivity to HMF.

An analogous set of experiments carried out in the presence of the various catalysts was performed with an initial concentration of fructose of 1 M. The obtained results are summarized in Figure 8B. By comparing the results reported in Figure 8A with those shown in Figure 8B, it can be observed an increase in the sugar conversion and a consequent decrease in selectivity to HMF when the initial concentration of fructose was increased from 0.3 to 1 M and, interestingly, also the yield to HMF increased. This occurred in all of the experiments, including the homogeneous reaction. The increased conversion of fructose observed by enhancing its initial concentration can be justified by considering the formation of some acidic species deriving from side reactions (see Scheme 1). The amount of these species is higher for a higher initial sugar concentration; consequently, a higher conversion is observed. Conversely, this occurrence induces a decrease in the selectivity versus the HMF formation. In this condition (1 M initial fructose concentration), the most acidic catalyst (Nb_2_O_5_) showed the highest conversion of fructose but at the same time the lowest selectivity, which was almost similar to that obtained in the homogeneous test. This fact indicates that the use of catalysts with lower acidity (the composites) reduces the occurrence of the side reactions and consequently increases the yield to HMF.

It is worth noting that, after the 3 h of experiment, the reaction medium was always strongly colored from orange to intense brownish. We observed, in agreement with the literature reports, that the color was more intense for the runs with a higher initial fructose concentration. The color is due to the formation of humins possessing various and complex polyfuranic molecular structures, whose structure strongly depends on the reaction conditions [18,61,62,63].

The dark solid residues obtained after the experiments carried out for 3 h with an initial concentration of fructose 1 M at 165 °C under the heterogeneous catalytic reaction were recovered and dried at 60 °C overnight. The complex nature of humins makes their characterization difficult; however, in the solid residues recovered after the reaction, some specific FTIR bands accounted for their presence. The FTIR spectra were recorded and the obtained results for some selected tests are shown in Figure S2 in the supporting information. Several authors have recorded analogous FTIR spectra, claiming the formation of cross-linked furan rings via intermolecular dehydration derived from HMF, as seen in Scheme 1 [18,61,62,63].

**Figure 9:**
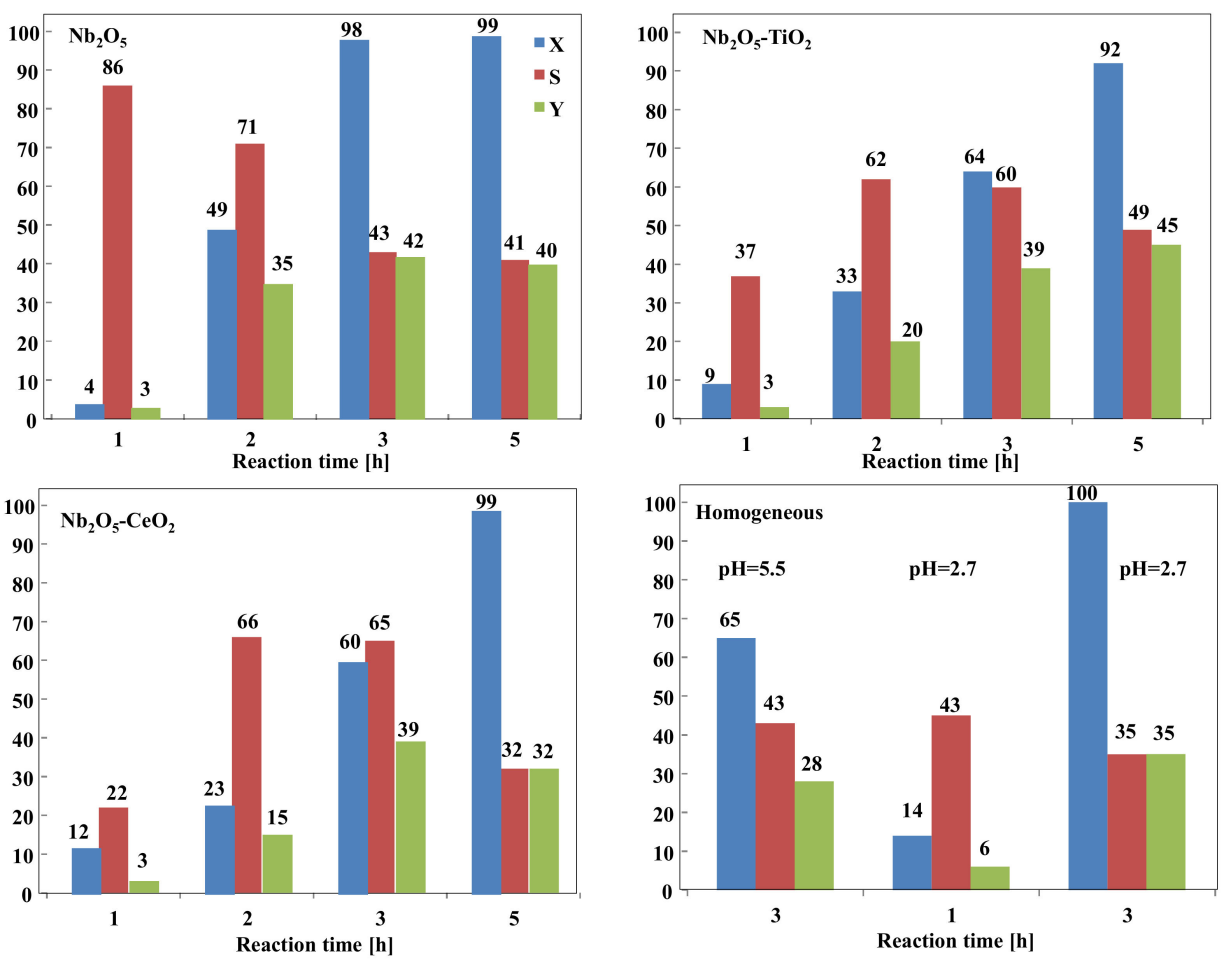
Fructose conversion (**X**), selectivity to HMF (**S**) and yield (**Y**) obtained in catalytic experiments carried out with an initial fructose concentration of 1 M at 165 °C in the presence of 6 mL of a suspension of 2 g·L^−1^ of heterogeneous catalysts or in homogeneous systems for reaction times ranging from 1 to 5 h.

In order to further verify the influence of the reaction time in the catalytic reaction, additional experiments were carried out in the presence of Nb_2_O_5_, Nb_2_O_5_-TiO_2_ and Nb_2_O_5_-CeO_2_ catalysts with an initial fructose concentration of 1 M at 165 °C. The catalytic results obtained for reaction times ranging from 1 to 5 h are reported in Figure 9. The results obtained in the homogeneous reaction system at natural pH and at pH = 2.7 (for H_2_SO_4_) are also displayed for the sake of comparison.

Fructose was slightly converted after 1 h of reaction both in the homogeneous and heterogeneous regime. The conversion increased by increasing the reaction time, reaching the maximum value at 5 h, although, in the presence of Nb_2_O_5_, 98% of conversion was reached after 3 h of reaction without a significant decrease in selectivity. In the presence of both Nb_2_O_5_-TiO_2_ and Nb_2_O_5_-CeO_2_, the conversion was higher after 5 h, but the selectivity decreased considerably with respect to what was obtained after 3 h, particularly in the presence of Nb_2_O_5_-CeO_2_. Interestingly, the yield was nearly maintained after 3 or 5 h of reaction. These results indicate that the maximum catalyst efficiency, in the current experimental conditions, was reached for 3 h of reaction time.

**Figure 10:**
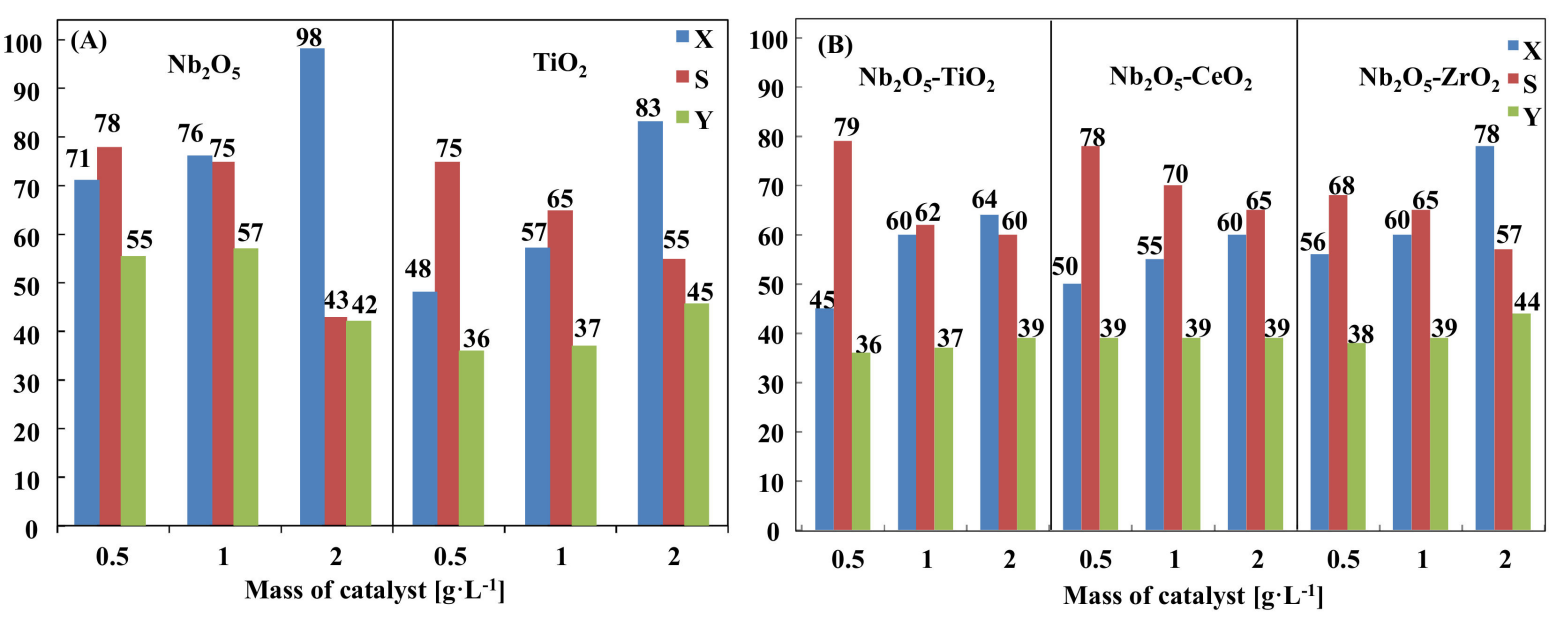
Fructose conversion (**X**), selectivity to HMF (**S**) and yield (**Y**) obtained in catalytic experiments carried out with an initial fructose concentration of 1 M at 165 °C for 3 h in the presence of 6 mL of a suspension of (**A**) Nb_2_O_5_ or TiO_2_ or (**B**) the composites as heterogeneous catalysts.

Eventually, some experiments, whose results are shown in Figure 10, were carried out by using a lower amount of catalyst in the reacting suspension.

As reported in Figure 10A,B, for all of the samples, the fructose conversion increased by increasing the catalyst amount but the selectivity versus HMF decreased. The lower amount of catalyst (0.5 and 1 g·L^−1^) produced always a higher selectivity to HMF, reaching the value of ca. 80% in the presence of 0.5 g·L^−1^ of Nb_2_O_5_, Nb_2_O_5_-TiO_2_ or Nb_2_O_5_-CeO_2_. It is important to note that the best compromise between high conversion and high selectivity, evidenced by the highest HMF yield of 55 and 57%, was obtained by using the most acidic sample, i.e., Nb_2_O_5_, in an amount corresponding to 0.5 and 1 g·L^−1^, respectively. It seems that the amount of catalytic acid sites is a very important parameter controlling the conversion of fructose to HMF, but it must be considered that, above a certain amount of these sites, although the transformation of fructose increases, the formation of side products also increases, reducing the yield to HMF. On the contrary, below this amount, the transformation of fructose is low, as the side product formation is also low. This fact indicates that there must be an optimum value of the amount of acid sites that, in this work, was likely reached by adding ca. 1 g·L^−1^ of Nb_2_O_5_ to the reacting suspension.

For the other samples, the increase in conversion observed by increasing the catalyst amount almost completely compensated for the decrease in the selectivity, giving rise to more or less the same yield, ca. 40%, for any amount of catalyst present in the reacting suspension. Only in the case of TiO_2_ and Nb_2_O_5_-ZrO_2_ materials, which are the most acidic catalysts after Nb_2_O_5_, an increase in the yield in HMF was observed when their amount inside the reaction suspension was 2 g·L^−1^ (ca. 45%). This finding seems to confirm that there is an optimal amount of acid sites needed to attain the highest possible yield in HMF. Consequently, since the catalysts have different quantities of acid sites, the amount of catalyst to be used in order to reach the maximum yield in HMF will be different. Obviously, acidity is not the only parameter to consider because also the specific surface area and the distribution of the pores can play an important role in the conversion of fructose to HMF, although, in this study, they seem less important with respect to the acidity of the system.

## 4. Conclusions

The catalytic dehydration of fructose to HMF was successfully performed in water suspensions of pristine Nb_2_O_5_ and composites containing Nb and Ti, Ce or Zr oxides. All the catalysts were characterized by powder X-ray diffraction, scanning electron microscopy, N_2_ physical adsorption, temperature-programmed desorption of NH_3_, infrared and Raman spectroscopy. Various experimental parameters, such as fructose initial concentration, volume of the reacting suspension, operation temperature, reaction time and amount of catalyst, were tuned to optimize the process. When the initial concentration of fructose increased from 0.3 to 1 M, an increase in the sugar conversion and a decrease in selectivity to HMF was observed. The conversion of fructose increased by increasing the reaction time from 1 to 5 h, with a decrease in the selectivity to HMF. The highest selectivity to HMF was reached in the presence of the lower amount of catalyst (0.5 g·L^−1^), but the best compromise between high conversion and high selectivity, evidenced by the highest HMF yield of 57%, was obtained by using the sample presenting the highest number of acidic sites, i.e., pristine Nb_2_O_5_, in an amount corresponding to 1 g·L^−1^. In particular, in this work, the best results (conversion 76% and selectivity to HMF 75% with a yield of 57%) were attained by using 1 g·L^−1^ of bare Nb_2_O_5_ as a catalyst for 3 h of reaction, working at a temperature of 165 °C in an autoclave by using 6 mL of 1 M fructose aqueous solution. These results are better than those obtained for analogous materials and conditions where a conversion of ca. 65% and a selectivity to HMF of ca. 30% were reached [32]. The acidity of the catalyst seems to be the most important factor influencing the conversion of fructose to HMF, and it is likely that there exists an optimal amount of acid sites for achieving the highest yield possible in HMF.

## Figures and Tables

**Table 1:** Chemical composition (nominal and EDX data) and textural properties of the prepared materials.

Catalyst	Nominal Nb/MetalRatio	EDX Nb/MetalRatio	Mean Crystallite Size ^a^		SSA (m^2^·g^−1^)	Cumulative Pore Volume (cm^3^·g^−1^)	Mean Pore Size (nm)
			Nb_2_O_5_ Phase	Oxide			
Nb_2_O_5_	-	-	14.2	-	54	0.17	10
Nb_2_O_5_-CeO_2_	0.5	1.0	n.d.	13.9	55	0.17	10
Nb_2_O_5_-TiO_2_	0.5	0.5	n.d.	17.0	38	0.07	5
Nb_2_O_5_-ZrO_2_	0.5	0.8	n.d.	14.7	50	0.06	4
TiO_2_	-	-	n.d.	11.6	73	0.21	9

^a^ Mean crystallite size was calculated by Scherrer equation.

**Table 2:** Total acidity of the prepared catalysts determined by NH_3_-TPD analysis.

Catalyst	(mmol NH_3_∙g·cat^−1^)
Nb_2_O_5_	0.076
Nb_2_O5-CeO_2_	0.039
Nb_2_O5-TiO_2_	0.038
Nb_2_O5-ZrO_2_	0.043
TiO_2_	0.055

**Table 3:** Conversion of fructose (X), selectivity (S) and yield (Y) to HMF obtained in catalytic experiments in homogeneous regime at natural pH (pH = 5.5), in the presence of H_2_SO_4_ (pH = 2.7) or Nb_2_O_5_ catalyst (2 g L^−1^), carried out for 3 h with an initial concentration of fructose equal to 1 M.

Catalyst.	t (°C)	X (%)	S (%)	Y (%)
Homogeneous pH = 5.5	165	65	43	28
Homogeneous pH = 2.7 for H_2_SO_4_	165	100	35	35
Nb_2_O_5_	165	98	43	42
Nb_2_O_5_	140	19	28	5
Nb_2_O_5_	100	0	0	0

## Data Availability

Not applicable.

## Supplementary Materials

The following are available online at https://www.mdpi.com/article/10.3390/nano11071821/s1, Figure S1: (A). XRD patterns of bare Nb_2_O_5_ and ICSD/JCPDS reference files for 2θ between 20–40°. (B). XRD patterns of Nb_2_O_5_-TiO_2_, TiO_2_ and anatase and rutile ICSD reference files for 2θ between 20–40°. (C). XRD patterns of Nb_2_O_5_-CeO_2_, Nb_2_O_5_-ZrO_2_ and the corresponding ICSD reference files for 2θ between 20–40°. Figure S2: FTIR spectra of the solid material recovered from the reactor after the fructose (1 M initial concentration) dehydration carried out for 3 h at 165 °C in presence of 2 g·L^−1^ of (a) Nb_2_O_5_-CeO_2_; (b) Nb_2_O_5_-ZrO_2_ and (c) Nb_2_O_5_.
