# Supplementary material for: Catalytic Dehydration of Fructose to 5-Hydroxymethylfurfural in Aqueous Medium over Nb2O5-Based Catalysts
Source: Nanomaterials (Basel). 2021 Jul 13;11(7):1821. doi: 10.3390/nano11071821 (PMC8308375; doi:10.3390/nano11071821)
Supplement: Supplementary file 1 [file nanomaterials-11-01821-s001.zip › nanomaterials-1272623-supplementary.pdf]

SUPPORTING INFORMATION

Catalytic dehydration of fructose to 5-hydroxymethylfurfural in aqueous medium over Nb_2O_5 based catalysts

Elisa I. García-López[a], Francesca Rita Pomilla[b], Bartolo Megna[b], Maria Luisa Testa[d], Leonarda Francesca Liotta[d], Giuseppe Marcì[b]

[a] Department of Biological, Chemical and Pharmaceutical Sciences and Technologies (STEBICEF), Università di Palermo, Viale delle Scienze, 90128 Palermo, Italy
[b] "Schiavello-Grillone" Photocatalysis Group. Dipartimento di Ingegneria, Università di Palermo, Viale delle Scienze, 90128 Palermo, Italy
[c] Istituto per Lo Studio dei Materiali Nanostrutturati (ISMN)-CNR, via Ugo La Malfa, 153, 90146, Palermo, Italy.
e-mail: giuseppe.marci@unipa.it

By comparing our experimental pattern of Nb_2O_5 with two reference files (ICDD n. 00-028-0317 and ICSD n.1840) a better correspondence with the orthorhombic structure was found as results by taking into account the diffraction peaks at 2θ angles in the 40-60° range (see Figure 1S (A)). According with the primary reference [1] signals related to the Nb_2O_5 phase (ICDD no. 00-028-0317) are reported up to 2θ of 70°.

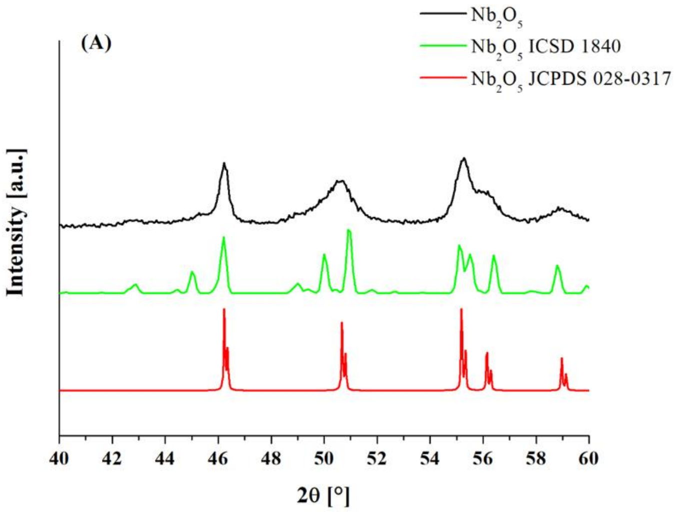

Figure S1 (A). XRD patterns of bare Nb_2O_5 and ICSD/JCPDS reference files for 2θ between 20-40 °.

The enlargement of the XRD pattern, for 2θ between 20-40 ° (see Figure S1 (B)), clearly evidences a shift to lower angles of the (101) from 25.4 ° in the pure TiO_2 to 25.15 ° in the Nb_2O_5-TiO_2 sample according with Nb insertion into the lattice of the anatase structure. A similar shift was observed looking at the 004 planes of the Nb doped titania along with a visible perturbation of peaks symmetry.

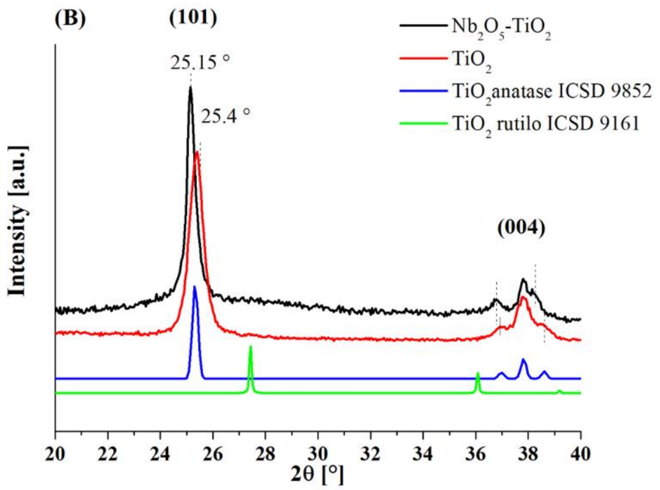

Figure S1 (B). XRD patterns of Nb_2O_5-TiO_2, TiO_2 and anatase and rutile ICSD reference files for 2θ between 20-40 °.

A detailed analysis of the XRD pattern, in the 2θ range 20-40° (see Figure S1 (C)) reveals a shift of the (111) peak of the CeO_2 fluorite structure from 28.55 ° to slightly higher angles, 28.69° that is indicative of an insertion of Nb(V), having ionic radius 0.74 Å smaller than 0.97 Å for eight coordinated Ce(IV) [2], into the ceria lattice. A similar shift was detected for the (200) peak.

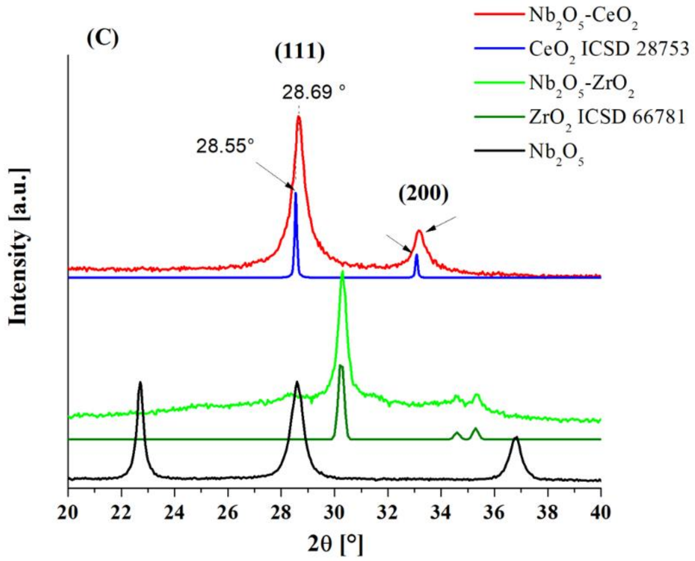

Figure S1 (C). XRD patterns of Nb_2O_5-CeO_2, Nb_2O_5-ZrO_2 and the corresponding ICSD reference files for 2θ between 20-40 °.

The results reported in Figure S2 are representative for all of the runs as long as the obtained spectra were very similar for all of the experiments. In Figure S2 (A) the broad band centred at 3400 cm^{-1} can be assigned to the O-H stretching of alcohols and/or carboxylic bonds and also due to the

presence of hydrogen bonds, the shoulder at ca. 3100 cm^{-1} can be attributed to stretching of C-H for furanics. The transition located at 2920 cm^{-1} can be attributed to the asymmetric C-H stretching of aliphatic groups. In Figure S2 (B), the transitions centred at ca. 1700 cm^{-1} can be attributed to C=O stretching from acids, aldehydes, and ketones, whereas the peaks at ca. 1620 cm^{-1} ascribed to the C=C stretching of the aromatic and furanic rings. The band located at ca. 1515 cm^{-1} is endorsed to stretching of the C-C in the furan rings, and the vibrations at 1290 and 1210 cm^{-1} can be attributed to C-O-C stretching whereas the band at 1020 cm^{-1} is the characteristic C-O deformation of the furan ring. The signals from C-H out-of-plane deformation at around 800 and 765 cm^{-1} could be ascribed to substituted furans [3]. The specific transitions at 1620, 1515 and 1395 cm^{-1} assigned to the characteristic stretching bands of the five-member furane ring, along with those at 800-700 cm^{-1} attributed to strong hydrogen wagging absorption of the five-membered ring indicate that the humins produced in these experiments are furan-rich structures, as evidenced by the specific five-member heteroaromatic ring transitions, in agreement with the literature. Several authors have recorder analogous FTIR spectra, claiming the formation of cross-linked furan rings via intermolecular dehydration derived from HMF as seen in Scheme 1 [3-6].

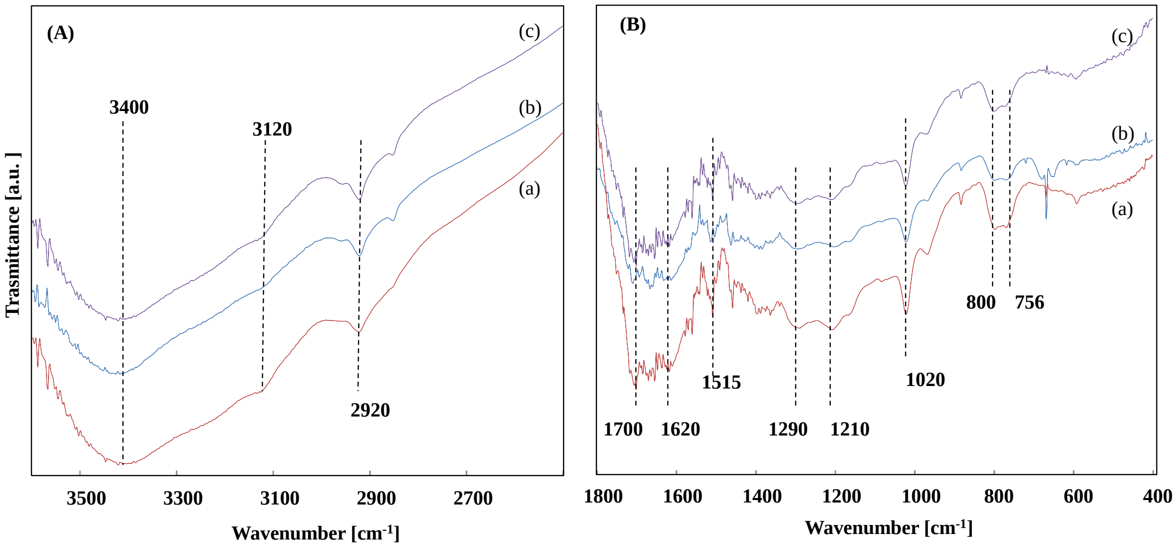

Figure S2. FTIR spectra of the solid material recovered from the reactor after the fructose (1 M initial concentration) dehydration carried out for 3 h at 165°C in presence of 2 $g \cdot L^{-1}$ of (a) Nb_2O_5-CeO_2; (b) Nb_2O_5-ZrO_2 and (c) Nb_2O_5.

REFERENCES

[1] L.K. Frevel, H.W. Rinn, Anal. Chem. (1955) 1329-1330.
[2] R.D. Shannon, Acta Cryst. A32 (1976) 751-767.
[3] I. van Zandvoort, Y. Wang, C. B. Rasrendra, E.R.H. van Eck, P. C.A. Bruijnincx, H.J. Heeres, B.M. Weckhuysen, ChemSusChem 6, (2013) 1745-1758
[4] Y. Nishimura, M. Suda, M.K. Kuroha, H. Kobayashi, K. Nakajima, A. Fukuoka, Carbohydr. Res. 486 (2019) 107826.
[5] S. K. Patil, J. Heltzel, C. R.F. Lund, Energy Fuels 26 (2012) 5281-5293.
[6] Z. Cheng, J.L. Everhart, G. Tsilomelekis, V. Nikolakis, Green Chem. 20 (2018) 997-1006
